# Complex Epidemiology of a Zoonotic Disease in a Culturally Diverse Region: Phylogeography of Rabies Virus in the Middle East

**DOI:** 10.1371/journal.pntd.0003569

**Published:** 2015-03-26

**Authors:** Daniel L. Horton, Lorraine M. McElhinney, Conrad M. Freuling, Denise A. Marston, Ashley C. Banyard, Hooman Goharrriz, Emma Wise, Andrew C. Breed, Greg Saturday, Jolanta Kolodziejek, Erika Zilahi, Muhannad F. Al-Kobaisi, Norbert Nowotny, Thomas Mueller, Anthony R. Fooks

**Affiliations:** 1 Animal and Plant Health Agency, New Haw, Addlestone, Surrey, United Kingdom; 2 School of Veterinary Medicine, University of Surrey, Guildford, United Kingdom; 3 Institute of Infection and Global Health, University of Liverpool, Liverpool, United Kingdom; 4 Institute of Molecular Virology and Cell Biology, Friedrich-Loeffler-Institut, Greifswald—Insel Riems, Germany; 5 Rocky Mountain Laboratories (NIAID, NIH), Hamilton, Montana, United States of America; 6 Formerly USAPHCR-Europe Laboratory Sciences, Veterinary Pathology, Landstuhl, Germany; 7 Institute of Virology, University of Veterinary Medicine Vienna, Vienna, Austria; 8 Department of Medical Microbiology and Immunology, College of Medicine and Health Sciences, United Arab Emirates University, Al Ain, United Arab Emirates; 9 Department of Microbiology and Immunology, College of Medicine and Health Sciences, Sultan Qaboos University, Muscat, Oman; The Global Alliance for Rabies Control, UNITED STATES

## Abstract

The Middle East is a culturally and politically diverse region at the gateway between Europe, Africa and Asia. Spatial dynamics of the fatal zoonotic disease rabies among countries of the Middle East and surrounding regions is poorly understood. An improved understanding of virus distribution is necessary to direct control methods. Previous studies have suggested regular trans-boundary movement, but have been unable to infer direction. Here we address these issues, by investigating the evolution of 183 rabies virus isolates collected from over 20 countries between 1972 and 2014. We have undertaken a discrete phylogeographic analysis on a subset of 139 samples to infer where and when movements of rabies have occurred. We provide evidence for four genetically distinct clades with separate origins currently circulating in the Middle East and surrounding countries. Introductions of these viruses have been followed by regular and multidirectional trans-boundary movements in some parts of the region, but relative isolation in others. There is evidence for minimal regular incursion of rabies from Central and Eastern Asia. These data support current initiatives for regional collaboration that are essential for rabies elimination.

## Author Summary

Despite being one of the oldest recognised infectious diseases, rabies continues to cause thousands of preventable human deaths per year. As a zoonotic disease, control of infection in the reservoir has been proven the most efficient route to reduction of human cases. In some regions, the epidemiology is well understood, with either dogs or wildlife known to be the primary reservoir and with little or no movement from, or into other regions. This is not the case in the Middle East, where rabies is underreported in animals and humans, there is little laboratory confirmation of infection, and the extent of rabies spread from country to country is not known. Previous studies have demonstrated trans-boundary movement of rabies but have been limited by a low number of available samples from some countries, and the direction of spread has been difficult to estimate. Here we use rabies virus partial genome sequences of 183 viruses from over 20 countries, combined with geographical and temporal information, to reconstruct the evolution of rabies viruses circulating in the Middle East. The results reveal an apparent barrier to spread between some regions but regular movement between others. These analyses will support policy on rabies control by indicating the relative importance of local control and animal movement restrictions when allocating resources.

## Introduction

Rabies is a fatal encephalitis caused by viruses in the genus *Lyssavirus* [1,2]. Although the majority of lyssavirus species are associated with bats, rabies virus (RABV) has successfully adapted to terrestrial carnivores on multiple occasions [3,4] and causes an estimated 70,000 deaths each year [5]. The majority of rabies cases in humans are caused by the bite of infected domestic dogs (*Canis lupus familiaris*), but rabies can persist in both domestic dog and wildlife reservoirs [6]. In addition to the morbidity and mortality burden, costs are incurred through the necessity for provision of post-exposure prophylaxis and surveillance in rabies endemic areas, leading to an annual global economic cost estimated at over 500 million dollars [7,8]. Concerted control efforts in many regions have demonstrated the feasibility of rabies elimination in carnivores [6,7,9]. These control efforts are dependent on local epidemiology of the disease which will vary from region to region, depending on differing ecological and sociological factors [10].

The Middle East is a politically diverse region with a rich cultural history, situated between Europe, Asia and Africa. This position, and the region’s political and cultural variety, have had implications for the control of trans-boundary diseases of animals such as Foot and Mouth Disease and zoonotic diseases such as Avian Influenza, Brucellosis and Middle East respiratory syndrome coronavirus (MERS-CoV) [11–14]. Economic restrictions, conflict and political instability can also affect surveillance for diseases, in addition to causing acute and unpredictable human or animal migration [15,16].

Recent reported annual incidences of human rabies in countries of the Middle East vary from 0.02 to 1.3 per million human population, with annual incidence of post exposure prophylaxis administration varying from 1700 to over 6000 per million [16,17]. These figures are heavily influenced by variation in surveillance and reporting in different countries, yet reflect an on-going burden of rabies in the region [16–18]. Although many countries in the Middle East collate and report human rabies cases, routine surveillance and reporting of animal rabies is less systematic, often relying on local awareness and resources with associated potential biases [16,17,19].

There are multiple historical reports of disease consistent with rabies throughout the history of civilisation in the Middle East (reviewed by [19] and [20]). Reconstruction of viral evolution using, molecular phylogenetics, suggests that the spread of RABV through human mediated dispersal from Europe is a dominant factor in the current epidemiology of rabies in the Middle East [4,21,22]. Dating this spread of a widely distributed lineage of RABVs, known as the ‘cosmopolitan’ lineage, using molecular clock analyses concurs with this scenario. The estimated date of divergence, 200–300 years ago, coincides with a period of increased human movements, increasing human population, and increasing domestic dog population associated with urbanisation in the 18^th^ century. Current epidemiological evidence also supports this, suggesting dog rabies predominates, at least in Jordan, Lebanon, Iraq and Iran, [16,17,19,23–27] and also remains a concern in some central Asian countries and in the Caucasus region [18,28]. However, the historical reports of disease consistent with rabies in the Middle East pre-date this relatively recent spread of the cosmopolitan lineage, and additional separate rabies lineages appear to be spreading in neighbouring regions which may also be circulating in the Middle East [16,18,29]. Throughout the Indian subcontinent and South East Asia, dogs are considered the principal reservoir of rabies, with a genetically distinct lineage of RABV predominating, indicating prolonged circulation without mixing from other regions [30–33]. In addition, more recently a lineage of RABVs related to those endemic in Arctic regions has emerged, and then become the dominant lineage in parts of South Asia [29,31,34,35]. Establishing whether rabies in the Middle East today is, for example, the result of ancient lineages that have persisted in the region, or the result of incursion from other areas is essential in targeting sustainable control methods.

Endemic dog rabies had been almost eliminated from Western Europe by the beginning of the 20^th^ century, but in the second half of the 20^th^ century an epidemic of fox (*Vulpes vulpes*) rabies spread through Europe, attributed to spill-over from dogs, most probably from more than one location in Eastern Europe [28,36–38]. With six wild canid species occupying the varied habitats in Western Russia and Eastern Europe, the epidemiology is complex [28]. The highest numbers of reported wildlife cases in Eastern Europe are in European red foxes and raccoon dogs (*Nyctereutes procyonoides*). In the Middle East rabies has been reported in foxes, but also in golden jackal (*Canis aureus*) and wolf (*Canis lupus lupus*) [17,39]. Control methods for dog rabies and wildlife rabies vary considerably in required knowledge base, stakeholder involvement and cost [9,10]. Therefore a better understanding of the epidemiology is a fundamental prerequisite to planning rabies control.

Previous phylogenetic studies of viruses detected in the Middle East have demonstrated probable trans-boundary movement of rabies, but have been unable to infer direction of movement and have been limited by numbers of samples available from some regions [16,22,27,36,40,41]. Here we have taken a broader and more comprehensive perspective on circulating RABV lineages in this particular region of the world by collecting RABV isolates from as many Middle East countries as possible as well as neighbouring regions. Through international collaborative efforts, we have obtained or generated sequence data for a panel of 183 RABVs spanning 40 years and from over 20 countries including previously un-sampled areas. We have also applied discrete phylogeographic techniques to infer migration events giving insight into rabies epidemiology in the region, with implications for control.

## Methods

### Ethics statement

No animals were used for this study. Brain samples were analysed that had previously been taken for diagnostic purposes, from animals that were already dead, with the permission of the local competent authority.

### Origin of viruses

A panel of 183 RABVs were selected for analysis from over 20 countries spanning the years between 1972 and 2014 (S1 Table). Sixty three virus sequences were derived in this study, with an additional 120 sequences derived in previous studies obtained from GenBank. Samples were collected as part of routine rabies sampling. This sampling will include rare systematic surveillance systems, but the majority of sequences will be from a laboratory-confirmed subset of ad-hoc reports of disease suspicion in either the public or animal health professionals. Samples include domestic dogs (ownerless, owned and free roaming, and owned and restricted) and wildlife.

### Viruses sequenced in this study

Virus sequences were derived from original clinical samples either in country of origin or in international reference laboratories (FLI Germany, APHA UK and VetmedUni, Vienna, Austria) on behalf of, and with permission of, local veterinary and public health authorities. Virus RNA was extracted from brain samples of rabid animals using TRIzol reagent, or using a guanidine based extraction kit (RNeasy, Qiagen) according to the manufacturer’s instructions. RNA was quantified by spectrophotometer and a 582 base pair region of the nucleoprotein (N) gene amplified by hemi-nested reverse transcriptase polymerase chain reaction (hn-RTPCR) as described previously (APHA and FLI) [42] or a combination of smaller RT-PCRs were used to generate a contiguous 948 base pair region of the nucleoprotein (Vienna, primer sequences are available on request). PCR products were purified (QiaQuick PCR Purification kit, Qiagen) and sequenced using chain-termination (Sanger) sequencing (Big Dye Sequencing kit, ABI). At least one forward and one reverse primer were used to generate a consensus sequence for each virus, which were visually checked for errors prior to alignment using the DNAStar package (Lasergene).

### Phylogenetic analysis

**Fig 1:**
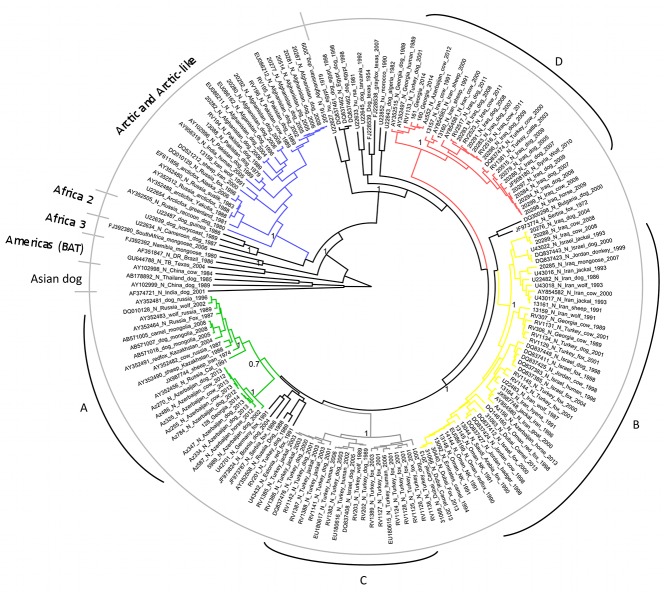
Maximum clade credibility tree from Bayesian reconstruction of 171 RABV partial nucleoprotein sequences, using a TN93 +G+I model of evolution with a strict molecular clock and Bayesian skyline prior, for 100 million iterations in BEAST (v1.8.0). The maximum clade credibility tree was chosen using TreeAnnotator(v1.8.0) and visualised using FigTree (v1.2). Posterior support is given at key nodes, and lineages are labelled according to previous studies [32] with the four main Middle East clades A-D labelled and coloured by region corresponding to Fig. 4.

Sequences generated in this study were combined with selected published RABV sequences to include, as far as possible, comprehensive cover of the Middle East, and representative sequences from surrounding regions. Sequences were aligned using ClustalX2 (version 1.2). The length of sequence available for analysis varied among isolates, but a 400 base pair region of the nucleoprotein gene was chosen for further analyses as it was common to 171 of the selected sequences. A phylogenetic analysis of all these 171 virus sequences was implemented using Bayesian Markov Chain Monte Carlo simulation in the BEAST packagev1.8.0 [43]. A TN93 nucleotide substitution model with rate variation and a proportion of invariant sites (Gamma+I) were determined to best fit the data using Akaike Information Criterion in MEGA 6.0. A relaxed and strict molecular clock model with either constant or Bayesian skyline population prior were used in Markov Chain Monte Carlo (MCMC) simulation for 100,000,000 iterations, sampling every 10,000 states to give effective sample sizes of over 200. Molecular clock and population coalescent models were compared using a modified Akaike information criterion (AICM) in Tracer v1.5 on a subset of 137 sequences, as described previously [44] (Table 1), and maximum clade credibility trees were annotated using TreeAnnotator (v1.8.0) after 10% of trees were discarded. The maximum clade credibility (MCC) trees were then visualised using Fig Tree (v1.4.0) (Fig. 1). A strict molecular clock and Bayesian skyline population prior gave the lowest AICM and were therefore used for the estimates of divergence dates. Neighbour-joining and maximum likelihood phylogenetic reconstructions were also undertaken on the data set in MEGA6 [45] for comparison with Bayesian reconstructions. A neighbour joining tree was derived from p-distances with 1000 bootstrap replicates and a maximum likelihood tree using the same TN93 +G +I evolutionary model as used in the Bayesian analyses, the Nearest-neighbour inference heuristic and 100 bootstrap replicates. NJ and ML trees were visualized in FigTree (v1.4.0).

**Table 1:** Assessment of model fit using a modified Akaike Information Criterion (AICM) for RABV partial nucleoprotein sequences.

Molecular clock	Population prior	AICM	Mean substitution rate (95% HPD)
Strict	Bayesian skyline	5478	3.88e-4 (2.75–5.01)
Strict	Constant	5546	4.06 e-4 (2.855–5.36)
Uncorrelated lognormal	Bayesian skyline	5552	4.09 e-4 (2.88–5.41)
Uncorrelated lognormal	Constant	5570	4.12 e-4 (2.94–5.39)

An MCMC chain length of 100 million iterations using 137 representative sequences in BEAST. AICM compared using Tracer (v1.6). Lower AICM values indicate better fit

An additional Bayesian MCMC phylogenetic analysis, with the same model parameters as above, was undertaken on a set of 59 sequences of 380 base pairs in length. These included 12 sequences from Oman and the United Arab Emirates which had only a 380 base pair region common to the other sequences, and were therefore not represented in the larger 400 base pair data set. These 12 were combined with 47 related sequences from the larger data set that were trimmed to 380 base pairs.

### Phylogeographic analysis

**Fig 2:**
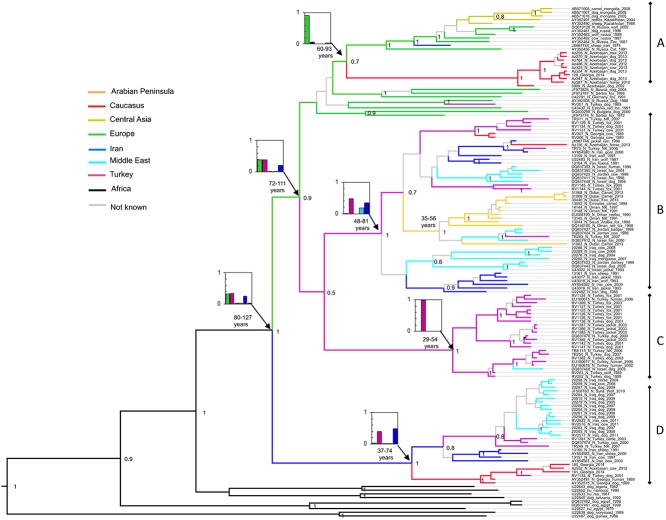
Discrete phylogeographic reconstruction of 139 RABV partial nucleoprotein sequences representing the ‘Cosmopolitan’ lineage, in the Middle East and surrounding regions. Model parameters are the same as Fig. 1, with the addition of viral origin location as a defined trait (Europe, Caucasus, Middle East, Arabian Peninsula, Turkey, Iran, Central Asia, and Africa). Branches are coloured by the most probable location of origin of the progeny node. Grey branches are used were the posterior support for the progeny node is low (below 50% probability). Inset are plots of relative probabilities the location of selected nodes coloured by location and the estimates of node ages (95% HPD years since 2014).

A discrete phylogeographic analysis was also undertaken on a subset of 139 samples (only viruses representing the ‘Cosmopolitan’ lineage). Samples were allocated to one of eight locations based on the country of isolation (Europe, Caucasus, Middle East, Arabian Peninsula, Iran, Central Asia, Africa, Turkey) (Table 2). These location groups were chosen to give approximately equal numbers of sequences per region and were based on geographical proximity of the recorded countries of origin. A data partition was generated from the location allocated to each sequence, meaning that each sequence is therefore identified by nucleotide sequence, date of isolation and location of origin. The same nucleotide substitution (TN93+G+I), strict molecular clock and Bayesian skyline population coalescent models were used. The probable locations of each ancestral node were then reconstructed in the ensuing Bayesian MCMC analysis and displayed on the maximum clade credibility tree annotated using TreeAnotator (v1.8.0) and visualised using FigTree (v1.4.0) (Fig. 2).

**Table 2:** Regional groupings for phylogeographic analyses.

REGION for phylogeographic analyses	COUNTRIES
EUROPE	Bosnia, Germany, Serbia, Ukraine, Bulgaria, Montenegro, Russia
CAUCASUS	Georgia, Azerbaijan
IRAN	Iran
TURKEY	Turkey
‘MIDDLE EAST’	Iraq, Jordan, Syria, Israel
CENTRAL ASIA	Mongolia, Kazakhstan
ARABIAN PENINSULA	Saudi Arabia, Oman, United Arab Emirates
AFRICA	South Africa, Tanzania, Guinea, Egypt, Morocco, Ivory Coast, Algeria

Country groupings do not reflect any political or cultural opinion and were determined using geographic location and sample numbers to maximise reliability of phylogeographic inferences

The strength of association between phylogenetic relationships and location of virus origin was assessed using an association index (AI) [46]. A null data set was created by randomisation of phylogeny and location data and an index of association between phylogeny and location in the observed data was compared to the equivalent from the null data set. The posterior set of trees produced by the Bayesian simulation was analysed with BaTS software (after burn-in was removed), to incorporate uncertainty in calculation of the AI [47]. Ratios between the AI for the data under test, and AI for the null data set give an indication of the strength of association between genetic and spatial information. Ratios approaching one suggest that genetically related viruses are spatially homogenous, whereas low AI ratios imply a strong association between location and phylogenetic relationships.

The number of trans-boundary movements or migration events suggested by the data were estimated using Markov jump counts [48], and the support for those migrations were assessed using a Bayesian stochastic search variable selection procedure (BSSVS), visualised using SPREAD [43], with Bayes factors greater than 90 taken as strong support.

### Reservoir species analysis

**Fig 3:**
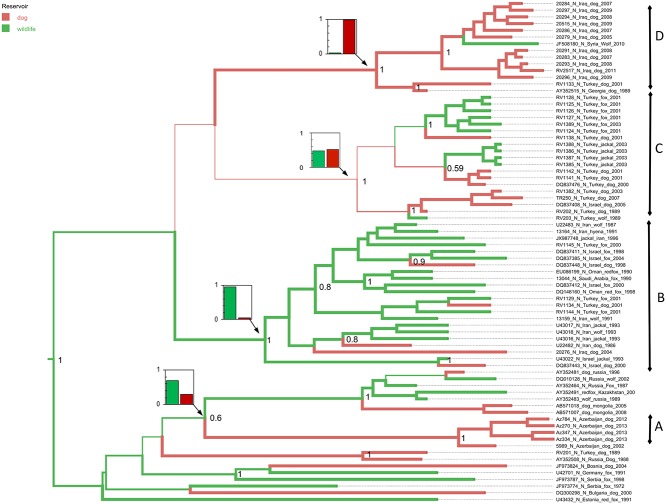
Maximum clade credibility tree using host species as a discrete trait, using only sequences from viruses detected in wild canids or dogs. Branches are coloured by the most probable host species (dogs or wildlife) of the progeny node, with inset plots of relative probability at key nodes.

A separate analysis was undertaken to infer the most probable reservoir for the ancestors of selected clades. In a similar manner to the phylogeographic analysis, each sequence was given a discrete trait corresponding to the species in which the virus sequences were detected. Due to the inherent uncertainty in whether the host a virus was detected in is the actual reservoir, all likely ‘dead-end’ hosts were removed. This stringent approach was taken to increase the reliability of the analyses at the expense of sample size, by only using sequences from domestic dogs (including ownerless and free-roaming dogs) (n = 38), and wild canids (n = 35). A TN93 G+I model of nucleotide substitution, a strict molecular clock and constant population size were used as model priors. The analysis was run for an MCMC chain length of 20,000,000 iterations and the chosen maximum clade credibility tree was annotated with TreeAnnotator (v1.8.0), visualised with FigTree (v1.4.0) and coloured by host species with the highest posterior probability (Fig. 3).

### Visualisation of circulating RABV lineages

**Fig 4:**
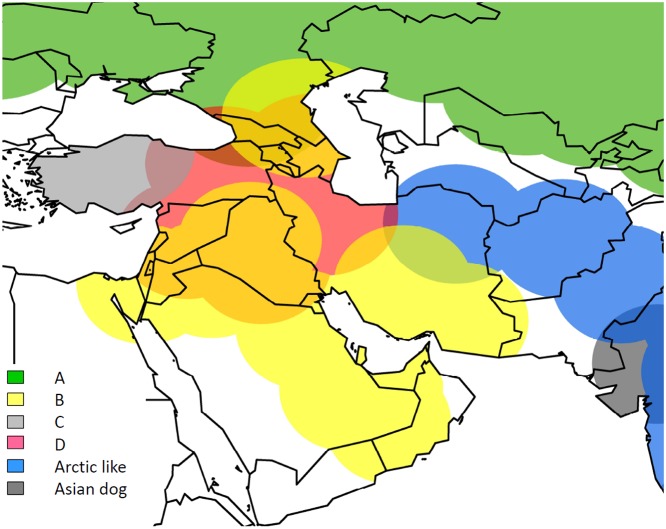
Map showing distribution of virus variants in Middle East, with clades coloured according to Fig. 1. Approximate distribution of each detected RABV lineage is displayed using ArcGis (ESRI ArcGis version 10.0).

The approximate distribution of each detected RABV lineage was visualised using ArcGis (ESRI ArcGis version 10.0) at the highest spatial resolution available for virus locations (Fig. 4).

## Results

### Inferred evolution of RABVs in the Middle East and Asia

These data support a clear and deeply rooted ancestral division of the represented RABVs into the well described ‘Cosmopolitan lineage’, and the previously characterised ‘Asian dog’ and ‘Arctic/Arctic-like’ lineages (Fig. 1). The division into these clades is conserved between trees inferred using NJ, ML and Bayesian methods (Figs. 1, S1, S2).

#### Pakistan and Afghanistan

There is strong support for genetic divergence between viruses from the ‘cosmopolitan’ lineage and other Asian viruses (Fig. 1). All the Asian viruses represented here share a common ancestor that is predicted to have occurred 60–300 years ago. All the viruses from Pakistan and Afghanistan form a monophyletic group within that clade, sharing a common ancestor that occurred 65–120 years ago (95%HPD). Viruses from Afghanistan and Pakistan are not genetically distinct from each other according to these analyses suggesting movement of infected animals between these countries. In stark contrast, there is relative genetic isolation from viruses detected in Iran, despite a border of over 1000 kilometres with Afghanistan and Pakistan. Only two sequences from Iran (‘DQ521212_sheep_Iran_2000’, and ‘13158_Iran_wolf_1991’) cluster with the Arctic-like lineage, and none of the ‘Cosmopolitan’ viruses have been detected in Afghanistan and Pakistan.

#### Middle East and Eastern Europe (‘Cosmopolitan’ lineage)

The evolution and spread of rabies west of this apparent barrier at 60 degrees longitude, corresponding to the Eastern border of Iran, is complex. These analyses suggest there are four well supported clades (A, B, C and D in Figs. 1, 4) currently or recently circulating in the Middle East and surrounding countries, which share a common ancestor predicted to have occurred 80–127 years ago:

The first, clade A (Fig. 2), has an ancestor that occurred in Europe (location probability 91%) within the past 90 years (95% HPD 60–93), and has then been introduced into the Caucasus region. The majority of the viruses from the Caucasus region (the majority represented here from Azerbaijan) occur in this clade. A single virus from Iran is also represented in this lineage, among viruses from European Russia. Inclusion of host identity on a reduced data set suggests that clade A had an ancestor in wildlife (probability >70%) (Fig. 3).

The second well supported clade (clade B, Fig. 2) has an ancestor which most probably occurred in Turkey or Iran (location posterior probabilities of 48% and 34%) within the last 80 years (95% HPD 48–81) from where it has then spread widely. Over the ensuing years there is evidence for three incursions of this clade into the Middle East, two into the Caucasus, and one into the Arabian Peninsula. All the viruses from the Arabian Peninsula lie in this lineage, suggesting a single introduction of rabies into the Arabian Peninsula 40 years ago (95% HPD 35–56). The host analysis strongly supports this clade being predominantly in wildlife, again with multiple introductions into dogs.

A third lineage (Clade C, Fig. 2) appears to have been introduced into Turkey from Europe and been maintained in Turkey with little evidence of trans-boundary movement for 50 years, with the exception of spread to Israel in 2005. This Turkish clade includes dog and wildlife isolates interspersed within three well supported sub-clades. One of these sub-clades has an ancestral virus with a high probability of originating in a dog, but the host origin of the others shares approximately equal probability of originating in dog or wildlife (Fig. 3).

The fourth lineage (Clade D, Fig. 2), has a well-supported ancestor that occurred in Iran or Turkey (location posterior probabilities 48% and 40%) within the last 75 years (95% HPD 37–74) and has then spread into the Caucasus, and the Middle East. The majority of the represented strains from Iraq, including the most recent strains from Baghdad in 2011, are therefore apparently due to incursion within the last 50 years. Viruses from dogs predominate in this lineage with the exception of a virus from a wolf in Syria (Fig. 3).

### Regional RABV diversity

#### Caucasus (countries included: Azerbaijan, Georgia)

As a geographical gateway between Europe and Asia, the Caucasus region unsurprisingly has had multiple introductions of rabies. These data support the occurrence of four introductions of rabies into the region, three of which have representative viruses detected within the past three years, implying at least short term maintenance in the region. Introductions appear to have come from multiple directions; Turkey, Iran and European Russia. Viruses from the Caucasus region represented here are all either from dogs or presumed spill-over hosts (domestic livestock and humans, which could be a consequence of spill-over from dogs or wildlife).

#### Iran

Viruses from Iran are represented in all four clades, A-D. The discrete phylogeographic analyses support introduction of the ‘Cosmopolitan’ lineage into Iran at least once, with subsequent persistence and seeding into other areas. Although the majority of virus sequences from Iran occur in this Cosmopolitan lineage, there are two sequences from Iran in the Arctic-like lineage, detected in a wolf in 1991 and a sheep in 2000. It is not clear whether these are separate incursions from other regions, or whether this indicates maintenance of the Arctic-like lineage in Iran. The majority of viruses sampled in Iran were detected in wildlife or presumed spill-over hosts despite the majority of reported human cases being dog mediated [49].

#### Turkey

Virus sequences from Turkey occur in three of the four well defined clades, with an additional virus sequence appearing as a probable independent introduction from Europe. The bulk of the viruses represented occur in one clade (clade C) but viruses from the other clades are circulating contemporaneously suggesting maintenance of distinct virus variants. Dog and wildlife samples from Turkey are interspersed in the tree, and within the dominant clade C there are sub-clades, which all also consist of dog and wildlife strains.

#### Middle East (countries included: Israel, Iraq, Jordan, and Syria)

Viruses from the Middle East also occur across three of the four clades, but with only one sequence (DQ837408) in clade C, suggested by these analyses as a probable introduction from Turkey. That single exception in clade C is highly similar to a cluster of 18 viruses isolated in Northern Israel between 2004 and 2006 which is likely to have moved from Turkey into Israel [27]. All other viruses from Israel and Jordan occur in clade B and appear to be introductions from Iran and the Arabian Peninsula. The host analysis and epidemiological data support the represented viruses from Israel and Jordan being largely wildlife derived, with evidence for spill over into dogs. In contrast, there is evidence that in Iraq there are viruses co-circulating both from this wildlife associated clade B and the dog associated clade D.

#### Arabian Peninsula (countries included: Saudi Arabia, United Arab Emirates, and Oman)

These data support a single, relatively recent, incursion into the Arabian Peninsula region, within clade B. All the viruses sampled from the Arabian Peninsula including recent samples from 2013, are descendant from a common ancestor, which probably also occurred in the Arabian Peninsula (location probability 92%) 40 years ago (95% HPD 35–56), suggesting endemic maintenance of infection in a local reservoir for the past 40 years. The majority of the virus sequences from the Arabian Peninsula are from wildlife, as is the case for the rest of clade B.

### Strength of association between geographic origin and phylogenetic relationships

The strength of association between spatial and genetic relationships was assessed using the Association index. When compared to the null data set, the sample of RABVs showed a strong association between geographical origin and phylogenetic relationships (AI ratio 2.11/11.52), p<0.01.

### Statistical support for migration events

Inference of non-negligible rates of migration through Bayesian stochastic search variable selection procedure (BSSVS) detected seven events within the study region, with Bayes Factor support over 90 (Table 3). These include migration between the Caucasus and other regions (Iran, Europe and Central Asia), and migration between Turkey and all neighbouring regions (Caucasus, Arabian Peninsula, Europe). Bayes Factor support for these migrations does not imply directionality, as a symmetrical network was assumed in the BSSVS procedure. However, combined with the discrete phylogeographic analysis these strongly support the picture of spread of rabies from Europe, Iran and Turkey into the other regions of the Middle East.

**Table 3:** Significant migration events among regional groupings in the Middle East.

Bayes Factor	Region 1	Region 2
413	Europe	Turkey
266	Caucasus	Central Asia
198	Turkey	Middle East
109	Turkey	Arabian Peninsula
103	Iran	Caucasus
102	Turkey	Caucasus
94	Europe	Caucasus

## Discussion

These analyses of a large number of RABV sequences from Europe, the Middle East and Asia provide a comprehensive overview of circulating RABV strains in domestic animals and wildlife, and show the geographic and temporal scale of trans-boundary movements of rabies within and between these regions. Previous studies have demonstrated likely spread in the region, but here we have been able to infer the direction of trans-boundary movements and make informed estimates of their frequency. These inferences have direct implications for rabies control policies and demonstrate that due to variation in patterns of rabies maintenance and spread a regional approach should be taken to reduce and eventually eliminate the disease.

The first written record of disease consistent with rabies associated with dogs comes from Mesopotamia, a region corresponding to modern day Iraq [16,20]. Rabies continues to be a significant public and animal health issue in the region almost 4000 years later. We found no evidence that currently circulating strains were direct descendants of virus ancestors that occurred in the region 4000 years ago. In contrast, these analyses support at least one introduction of rabies from Europe with subsequent spread, albeit on a markedly different timescale, in the last 150 years. Indeed, estimates of the origin of all RABVs currently circulating in non-flying mammals are as recent as 1000 years ago. Our estimates of viral evolutionary rates in the partial nucleoprotein gene sequence concur with these previous analyses [4,32,36,50]. It is possible that ancestors of these ancient viruses are still circulating undetected, though a much more parsimonious explanation is that these ancient RABVs have been replaced by contemporary viruses [4,51]. A further consideration is that there is likely to be variability in selection pressures over time that nucleotide substitution models are unable to properly account for. This variability can lead to gross underestimates of the origins of viral clades which could explain the discrepancy between the historical records and molecular dating for RABV origins [52].

There is a clear and strongly supported distinction between those viruses circulating in Pakistan and Afghanistan (Arctic-Like 1b), and those further west. Inclusion of recent isolates from Afghanistan add to previous studies [29,31,53] demonstrating that the dominant lineage in Pakistan and Afghanistan is closely related to Arctic RABVs. With the exception of two viruses detected in Iran also from the Arctic-like lineage, there is an apparent barrier to spread of the Arctic-like lineage at approximately 60 degrees longitude, corresponding to the Iranian border. Similarly, the Cosmopolitan lineage viruses appear to have spread widely to the west of 60 degrees longitude but not to the East, and it is currently unclear why. There is evidence that the RABV within the ‘Cosmopolitan’ lineage that are closely related to Chinese street strains are due to poorly attenuated vaccines [54]. Natural barriers such as high mountain ranges are unlikely to serve as a complete explanation here considering the fact that Arctic-like lineages appear able to cross the Himalaya [29,53]. The east of Iran is much less densely populated than the west, with large tracts of uninhabited land. With good evidence for human mediated dispersal of dog rabies in other regions [48], human movements are likely to play a role in the Middle East. The nomadic pastoralist communities in parts of Iran and Central Asia that keep ‘choupan’ watch dogs [55] would be a potential route of trans-boundary movement of rabies, and also wildlife such as jackal (*Canis aureus*) and wolf (*Canis lupus*) are able to move freely across borders. Whether the Arctic-like lineage now dominant in Afghanistan, Pakistan and India continues to spread remains to be seen, but this apparent barrier to recent rabies spread between Iran and Afghanistan/Pakistan could be extremely important and deserves further investigation.

In contrast with the apparent barrier to eastward spread at 60 degrees longitude, we have demonstrated significant support for movement of rabies among countries to the west of Iran. Notably, at least one of the clades of recent or currently circulating viruses sampled in this study (Clade D) had an ancestor that most probably occurred in Iran. Dispersal of dog rabies has been strongly associated with human movements [48,56]. The history of human migration in the Middle East is complex, dynamic and has been affected by civil unrest and conflict leading to poor reporting or recording of human movements [57,58]. In addition, comparatively little quantitative data are available for dog demographics. These two knowledge gaps limit understanding spread and therefore planning control strategies for dog rabies [59,60]. However, considering that the overwhelming majority of human cases are caused by bites from domestic dogs, systematic regular dog vaccination campaigns are currently still required to prevent human cases.

Wildlife rabies presents a complex challenge, as demonstrated by the expensive and prolonged reduction of fox rabies in Western Europe through oral vaccination with live attenuated rabies vaccines [9]. In addition, as we have demonstrated here, wildlife and dog rabies are not as distinct as could be expected from this Western European experience [9,61,62]. For example, lineage B is wildlife-associated, but descendants of that lineage are now in dogs in Turkey and Azerbaijan. It is not possible to distinguish reliably with these data whether the samples are the result of repeated spill over from a wildlife reservoir or whether they represent maintenance of rabies in dogs. This is a critical question in rabies control, as demonstrated in parts of Africa where elimination of rabies in domestic dogs has led to the reduction of rabies in wildlife [63]. Both wildlife and dog rabies continue to be reported in Azerbaijan and Turkey, and epidemiological studies in Turkey and Israel strongly support spill over from wildlife [27,64–67]. Although control of dogs remains a priority from a public health perspective, eliminating rabies will require control in all reservoirs. Fortunately, with the advent of rapid and affordable sequencing [68], and increased diagnostic testing and surveillance capacity in resource limited countries, it should be possible to resolve this important issue and establish whether there is independent maintenance of rabies in wildlife in these countries [69].

Historical records of rabies in the Middle East have often involved infection in gray wolves (*Canis lupus*) including an incident of one wolf infecting over 100 people in Adalia, Turkey in 1850, and another significant event with multiple human fatalities in Iran in 1954 [70,71]. Wolves are not considered a natural reservoir due to their social structure and demography [72] but are an important vector of rabies to humans. For example, wolf bites historically featured disproportionately as the cause of human rabies cases in Russia, responsible for up to 56% of human cases toward the end of the 19^th^ century [73]. The large size and ferocity of rabid wolves may lead to a disproportionate number of people and domestic animals affected, but also increase the likelihood of the event being detected and reported. With evidence of recent interbreeding between wolves and shepherd dogs in the Caucasus, and now the evidence for a dog associated strain in a wolf in Syria, there is potential for more complex reservoir dynamics [74]. Again, continued improvement of systematic surveillance and international reporting will help resolve these questions [16–18,39].

As with all studies using routine sampling for rabies, these data are limited by sporadic and inconsistent sample selection. This ad-hoc nature of most animal rabies sampling predisposes to bias towards animals of economic value, or those most likely to come into contact with humans. We cannot rule out the possibility of failing to sample all currently circulating lineages, nor account for the effect on the phylogeographic analysis of lineages that have become extinct. For example, epidemiological data supports the elimination of dog rabies from Europe in the latter part of the 20^th^ century and those viruses are not represented here. The number and host species of samples from each country must also not be over-interpreted due to possible bias from differences in sampling strategy and the relative accessibility of samples from domestic animals or wildlife. For the discrete phylogeographic and reservoir host analyses, data were deliberately allocated into groups (regions, dog/wildlife) to improve the power of the analyses, at the expense of resolution. The countries were grouped based on geographic location and to give approximately equal numbers of sequences per group. Limited location data were available for many of the sequences (country level only) making continuous phylogeography unsuitable here, but if further location data were available the details of rabies dispersal would lend themselves to quantitative phylogeographic analyses. Considering this fact the distribution of RABV lineages identified in this study as shown in Fig. 4 are an approximation based on limited sequence location data. Nevertheless, mapping of the lineages allowed a more comprehensive view on the presumed geographic distribution in the Middle East that would be obtained from phylogenetic trees alone (Figs. 1, 2). Our analyses were limited by available sequence length, with many of the sequences from historical samples having only a partial nucleoprotein gene sequence available and no original sample available for further sequencing. Comparison of relationships to those inferred using other regions such as the G-L intergenic region used in previous studies was therefore not possible. While this may restrict resolution, the analyses produced phylogenetic trees with strong posterior support for key nodes allowing reliable interpretation of those nodes, and major differences in relationships would not be expected, based on results from previous studies [38,75].

Dogs are responsible for the majority of human rabies cases; something that has been demonstrated conclusively in other regions, and where investigated, also in the Middle East [16,17,19,23–27,60]. Therefore adequate vaccine coverage in owned dogs should remain a priority [76]. However, these data show regular host switching and repeated trans-boundary movement of rabies demonstrating that long term control will require a more detailed understanding of dog demographics and human movements, a high resolution assessment of the role of wildlife and cross-species transmission in the maintenance of rabies in the region and a regional approach to rabies control. Current international collaborative efforts in the region tasked with improving surveillance and international reporting will facilitate achieving these aims [17,18,39].

## Supporting Information

S1 Table Details of sequences used in this study. (DOC)

S1 Fig A maximum likelihood phylogenetic tree of 171 rabies virus partial nucleoprotein gene sequences, inferred using the same TN93 +G +I evolutionary model as used in the Bayesian analyses, the Nearest-neighbour inference heuristic and 100 bootstrap replicates (MEGA6). Bootstrap values are shown at selected key nodes.
(TIF)

S2 Fig Neighbour joining tree of 171 partial nucleoprotein rabies gene sequences, derived from p-distances with 1000 bootstrap replicates (MEGA6). Boostrap values are shown at selected key nodes.
(TIF)
