# Supplementary material for: Complex Epidemiology of a Zoonotic Disease in a Culturally Diverse Region: Phylogeography of Rabies Virus in the Middle East
Source: PLoS Negl Trop Dis. 2015 Mar 26;9(3):e0003569. doi: 10.1371/journal.pntd.0003569 (PMC4374968; doi:10.1371/journal.pntd.0003569)
Supplement: S1 Table — (DOC) [file pntd.0003569.s001.doc]

Table S1 sequences used in this study

| **Origin** | **Year** | **Genbank #** | **ID** | **Species** | **Details/reference** |
| --- | --- | --- | --- | --- | --- |
| **Afganistan** | 1996 | EU086162 | na | dog | Bourhy et al, 2008 |
| **Afghanistan** | 2004 | EU086211 | 04029AFG | dog | Bourhy et al, 2008 |
| **Afghanistan** | 2002 | EU086212 | 02052AFG | dog | Bourhy et al, 2008 |
| **Afghanistan** | 2005 | KP723575 | 20277 | dog | This study(FLI) |
| **Afghanistan** | 2006 | KP723576 | 20280 | dog | This study(FLI) |
| **Afghanistan** | 2006 | KP723577 | 20281 | dog | This study(FLI) |
| **Afghanistan** | 2006 | KP723578 | 20282 | dog | This study(FLI) |
| **Afghanistan** | 2008 | KP723579 | 20287 | dog | This study(FLI) |
| **Afghanistan** | 2008 | KP723580 | 20292 | dog | This study(FLI) |
| **Afghanistan** | 2005 | KP723581 | 20514 | pig | This study(FLI) |
| **Afghanistan** | 2009 | KP723582 | 20516 | dog | This study(FLI) |
| **Alaska** | 2006 | EF611856 | na | arctic fox | Kuzmin et al., 2008 |
| **Algeria** | 1982 | U22643 | na | dog | Kissi et al., 1995 |
| **Azerbaijan** | 2002 | KP723583 | 5989 | dog | This study(FLI) |
| **Azerbaijan** | 2013 | KJ645921 | Az156 | horse | Zeynalova et al,2014 |
| **Azerbaijan** | 2013 | KJ645922 | Az255 | cow | Zeynalova et al,2014 |
| **Azerbaijan** | 2013 | KJ645923 | Az270 | dog | Zeynalova et al,2014 |
| **Azerbaijan** | 2013 | KJ645924 | Az325 | cow | Zeynalova et al,2014 |
| **Azerbaijan** | 2013 | KJ645925 | Az334 | dog | Zeynalova et al,2014 |
| **Azerbaijan** | 2013 | KJ645926 | Az347 | dog | Zeynalova et al,2014 |
| **Azerbaijan** | 2012 | KJ645927 | Az486 | cow | Zeynalova et al,2014 |
| **Azerbaijan** | 2012 | KJ645928 | Az552 | cow | Zeynalova et al,2014 |
| **Azerbaijan** | 2012 | KJ645929 | Az587 | horse | Zeynalova et al,2014 |
| **Azerbaijan** | 2012 | KJ645930 | Az784 | dog | Zeynalova et al,2014 |
| **Bosnia** | 2004 | JF973824 | na | dog | McElhinney et al., 2011 |
| **Brazil** | 1986 | AF351847 | na | bat | Nadin-Davis et al., 2001 |
| **Bulgaria** | 2000 | DQ300298 | na | dog | Johnson et al 2007 |
| **Cameroon** | 1987 | U22634 | na | dog | Kissi et al., 1995 |
| **China** | 1984 | AY102998 | na | cow | Smith et al., 2004 |
| **China** | 1989 | AY102999 | na | dog | Smith et al., 2005 |
| **Dubai** | 2013 | KP723584 | 30440 | fox | This study(FLI) |
| **Dubai** | 2013 | KP723585 | 31063 | camel | This study(FLI) |
| **Dubai** | 2013 | KP723586 | 31066 | camel | This study(FLI) |
| **Dubai** | 2013 | KP723587 | 31068 | camel | This study(FLI) |
| **Egypt** | 1998 | DQ837461 | na | dog | David et al., 2009 |
| **Egypt** | 1998 | DQ837462 | na | dog | David et al., 2009 |
| **Egypt** | 1998 | DQ837462 | na | dog | David et al., 2009 |
| **Egypt** | 1979 | U22627 | na | human | Kissi et al., 1995 |
| **Emirates** | 1994 | KP723588 | 13092 | camel | This study(FLI) |
| **Estonia** | 1991 | U43432 | 9342EST | raccoon dog | Kissi et al., 1995 |
| **Georgia** | 1989 | AY352497 | RV308 | human | Kuzmin et al., 2004 |
| **Georgia** | 1989 | AY352515 | RV305 | dog | Kuzmin et al., 2004 |
| **Georgia** | 1989 | AY352516 | RV307 | cow | Kuzmin et al., 2004 |
| **Georgia** | 1989 | DQ317519 | RV306 | cow | Kuzmin et al., 2004 |
| **Georgia** | 2014 | KP723589 | 160 | na | This study (APHA) |
| **Georgia** | 2014 | KP723590 | 161 | na | This study (APHA) |
| **Georgia** | 2014 | KP723591 | 128 | na | This study (APHA) |
| **Germany** | 1991 | U42701 | na | fox | Bourhy et al.,direct submission |
| **Greenland** | 1981 | U22654 | na | Arctic fox | Kissi et al., 1995 |
| **Guinea** | 1986 | U22487 | na | dog | Kissi et al., 1995 |
| **India** | 2001 | AF374721 | na | dog | Jayakumar et al., 2004 |
| **India** | 2005 | AY956319 | na | human | Pfefferle,S. direct submission |
| **Iran** | 2000 | AY854580 | V685 | goat | Nadin-Davis et al., 2003 |
| **Iran** | 2000 | AY854581 | V686 | cow | Nadin-Davis et al., 2003 |
| **Iran** | 2000 | AY854582 | V699 | cow | Nadin-Davis et al., 2003 |
| **Iran** | 2000 | AY854583 | V703 | sheep | Nadin-Davis et al., 2003 |
| **Iran** | 2000 | DQ521212 | na | sheep | Nadin-Davis et al., Direct submission |
| **Iran** | 1974 | JX987744 | na | sheep | Pant et al., 2013 |
| **Iran** | 1996 | JX987748 | na | jackal | Pant et al., 2013 |
| **Iran** | 1991 | KP723592 | 13157 | cow | This study(FLI) |
| **Iran** | 1991 | KP723593 | 13158 | wolf | This study(FLI) |
| **Iran** | 1991 | KP723594 | 13159 | wolf | This study(FLI) |
| **Iran** | 1991 | KP723595 | 13160 | sheep | This study(FLI) |
| **Iran** | 1991 | KP723596 | 13161 | sheep | This study(FLI) |
| **Iran** | 1991 | KP723597 | 13164 | hyena | This study(FLI) |
| **Iran** | 1986 | U22482 | 8681IRA | dog | Kissi et al., 1995 |
| **Iran** | 1987 | U22483 | 8702IRA | wolf | Kissi et al., 1995 |
| **Iran** | 1993 | U43016 | 9308IRA | jackal | Bourhy et al., 1999 |
| **Iran** | 1993 | U43017 | 9309IRA | Jackal | Bourhy et al., 1999 |
| **Iran** | 1993 | U43018 | 9320IRA | wolf | Bourhy et al., 1999 |
| **Iraq** | 2011 | JX5241277 | RV2517 | dog | Horton et al., 2013 |
| **Iraq** | 2011 | JX524176 | RV2516 | cow | Horton et al., 2013 |
| **Iraq** | 2011 | JX524178 | RV2523 | cow | Horton et al., 2013 |
| **Iraq** | 2004 | KP723598 | 20276 | dog | This study(FLI) |
| **Iraq** | 2005 | KP723599 | 20279 | dog | This study(FLI) |
| **Iraq** | 2007 | KP723600 | 20283 | dog | This study(FLI) |
| **Iraq** | 2007 | KP723601 | 20284 | dog | This study(FLI) |
| **Iraq** | 2007 | KP723602 | 20285 | mongoose | This study(FLI) |
| **Iraq** | 2007 | KP723603 | 20286 | dog | This study(FLI) |
| **Iraq** | 2008 | KP723604 | 20288 | cow | This study(FLI) |
| **Iraq** | 2008 | KP723605 | 20289 | cow | This study(FLI) |
| **Iraq** | 2008 | KP723606 | 20291 | dog | This study(FLI) |
| **Iraq** | 2008 | KP723607 | 20293 | dog | This study(FLI) |
| **Iraq** | 2008 | KP723608 | 20294 | dog | This study(FLI) |
| **Iraq** | 2009 | KP723609 | 20296 | dog | This study(FLI) |
| **Iraq** | 2009 | KP723610 | 20297 | dog | This study(FLI) |
| **Iraq** | 2009 | KP723611 | 20298 | horse | This study(FLI) |
| **Iraq** | 2008 | KP723612 | 20299 | cow | This study(FLI) |
| **Iraq** | 2009 | KP723613 | 20515 | dog | This study(FLI) |
| **Israel** | 1996 | DQ837383 | na | human | David et al., 2009 |
| **Israel** | 2004 | DQ837385 | na | fox | David et al., 2009 |
| **Israel** | 2005 | DQ837408 | na | dog | David et al., 2009 |
| **Israel** | 1998 | DQ837411 | na | fox | David et al., 2009 |
| **Israel** | 2000 | DQ837412 | na | fox | David et al., 2009 |
| **Israel** | 2000 | DQ837443 | na | dog | David et al., 2009 |
| **Israel** | 1998 | DQ837448 | na | dog | David et al., 2009 |
| **Israel** | 1993 | U43022 | 9332ISR | jackal | Bourhy et al., 1999 |
| **Ivory coast** | 1989 | U22639 | na | dog | Kissi et al., 1995 |
| **Jordan** | 1999 | DQ837423 | donkey/J2/1999 | donkey | David et al., 2009 |
| **Jordan** | 1998 | DQ837424 | cow/J3/1998 | cow | David et al., 2009 |
| **Jordan** | 1998 | DQ837425 | cow/J4/1998 | cow | David et al., 2009 |
| **Jordan** | 1998 | DQ837427 | badger/J6/1998 | badger | David et al., 2009 |
| **Kazakhstan** | 1988 | AY352490 | na | sheep | Kuzmin et al., 2004 |
| **Kazakhstan** | 2004 | AY352491 | na | Red fox | Kuzmin et al., 2004 |
| **Mongolia** | 2008 | AB571005 | na | camel | Boldbaatar et al., 2010 |
| **Mongolia** | 2008 | AB571007 | na | dog | Boldbaatar et al., 2011 |
| **Mongolia** | 2005 | AB571018 | na | dog | Boldbaatar et al., 2012 |
| **Morocco** | 1990 | U22852 | na | human | Kissi et al., 1995 |
| **Namibia** | 1980 | FJ392392 | na | mongoose | Van Zyl et al., 2010 |
| **Oman** | 1998 | DQ146160 | RV643 | Red fox | direct submission |
| **Oman** | 1990 | EU086199 | na | Red fox | Bourhy et al, 2008 |
| **Oman** | 1991 | KP723614 | 13145 | na | This study(FLI) |
| **Oman** | 1991 | KP723615 | 13148 | na | This study(FLI) |
| **Oman** | 1991 | KP723616 | 14144 | na | This study(FLI) |
| **Oman** | 2002 | KP723617 | RV I | Fox | This study (Vienna) |
| **Oman** | 2002 | KP723618 | RVII | fox | This study (Vienna) |
| **Oman** | 2002 | KP723619 | RV III | camel | This study (Vienna) |
| **Oman** | 2004 | KP723620 | RV IX | cow | This study (Vienna) |
| **Oman** | 2003 | KP723621 | RV V | cow | This study (Vienna) |
| **Oman** | 2003 | KP723622 | RV VII | sheep | This study (Vienna) |
| **Oman** | 2003 | KP723623 | RV VIII | goat | This study (Vienna) |
| **Oman** | 2004 | KP723624 | RV X | cow | This study (Vienna) |
| **Oman** | 2004 | KP723625 | RV XI | camel | This study (Vienna) |
| **Pakistan** | 1989 | AY102996 | RV193 | dog | Smith et al., 2003 |
| **Pakistan** | 1979 | KP723626 | 13088 | dog | This study(FLI) |
| **Pakistan** | 1989 | KP723627 | RV194 | dog | This study(APHA) |
| **Pakistan** | 1989 | KP723628 | RV195 | dog | This study(APHA) |
| **Pakistan** | 1989 | KP723629 | RV196 | cow | This study(APHA) |
| **South Africa** | 1981 | U22633 | na | human | Kissi et al., 1995 |
| **Russia** | 1991 | AY352456 | na | cat | Kuzmin et al., 2004 |
| **Russia** | 1987 | AY352464 | na | fox | Kuzmin et al., 2004 |
| **Russia** | 1983 | AY352480 | na | suslik | Kuzmin et al., 2004 |
| **Russia** | 1996 | AY352481 | na | dog | Kuzmin et al., 2004 |
| **Russia** | 1987 | AY352482 | na | cow | Kuzmin et al., 2004 |
| **Russia** | 1989 | AY352483 | na | wolf | Kuzmin et al., 2004 |
| **Russia** | 1980 | AY352505 | na | raccoon dog | Kuzmin et al., 2004 |
| **Russia** | 1988 | AY352508 | na | dog | Kuzmin et al., 2004 |
| **Russia** | 1988 | AY352512 | na | Arctic fox | Kuzmin et al., 2004 |
| **Russia** | 2002 | DQ010128 | RV1336 | wolf | Mansfield et al., 2006 |
| **Russia** | 1996 | DQ010129 | na | fox | Mansfield et al., 2007 |
| **Saudi Arabia** | 1990 | KP723630 | 13044 | fox | This study(FLI) |
| **Serbia** | 1972 | JF973774 | na | fox | McElhinney et al., 2011 |
| **Serbia** | 1998 | JF973787 | na | fox | McElhinney et al., 2011 |
| **South Africa** | 2006 | FJ392380 | na | mongoose | Van Zyl et al., 2010 |
| **Syria** | 2010 | JF508180 | na | wolf | Johnson et al., direct submission |
| **Tanzania** | 1992 | U22645 | na | dog | Kissi et al., 1995 |
| **Texas** | 2007 | FJ228538 | na | gray fox | Velasco-Villa et al., 2008 |
| **Texas** | 1954 | FJ228539 | na | dog | Velasco-Villa et al., 2009 |
| **Texas** | 2004 | GU644788 | na | bat | Streiker et al., 2010 |
| **Thailand** | 1985 | AB178892 | THA1013 | dog | Arai, 2004 |
| **Turkey** | 1989 | AY091608 | RV202 | dog | Johnson et al., 2003 |
| **Turkey** | 1989 | AY091609 | RV203 | wolf | Johnson et al., 2003 |
| **Turkey** | 2001 | AY091610 | RV1124 | fox | Johnson et al., 2003 |
| **Turkey** | 2001 | AY091611 | RV1125 | fox | Johnson et al., 2003 |
| **Turkey** | 2001 | AY091612 | RV1126 | fox | Johnson et al., 2003 |
| **Turkey** | 2001 | AY091613 | RV1127 | fox | Johnson et al., 2003 |
| **Turkey** | 2001 | AY091614 | RV1128 | fox | Johnson et al., 2003 |
| **Turkey** | 2001 | AY091615 | RV1129 | fox | Johnson et al., 2003 |
| **Turkey** | 2001 | AY091617 | RV1131 | cow | Johnson et al., 2003 |
| **Turkey** | 2001 | AY091619 | RV1133 | dog | Johnson et al., 2003 |
| **Turkey** | 2001 | AY091620 | RV1134 | dog | Johnson et al., 2003 |
| **Turkey** | 2001 | AY091624 | RV1141 | dog | Johnson et al., 2003 |
| **Turkey** | 2003 | AY536254 | RV1385 | jackal | Johnson et al. ,2006 |
| **Turkey** | 2003 | AY536255 | RV1386 | jackal | Johnson et al. ,2006 |
| **Turkey** | 2003 | AY536256 | RV1387 | jackal | Johnson et al. ,2006 |
| **Turkey** | 2003 | AY536257 | RV1388 | jackal | Johnson et al. ,2006 |
| **Turkey** | 2003 | AY536258 | RV1389 | fox | Johnson et al. ,2006 |
| **Turkey** | 2001 | AY538776 | RV1142 | dog | Johnson et al. ,2006 |
| **Turkey** | 2003 | AY956352 | RV1381 | cow | Johnson et al., 2006 |
| **Turkey** | 2000 | DQ837474 | T1 | cow | David et al., 2009 |
| **Turkey** | 2000 | DQ837476 | T3 | dog | David et al., 2009 |
| **Turkey** | 1989 | EU180613 | RV201 | dog | Un et al., 2009 |
| **Turkey** | 2006 | EU180615 | TR3 | human | Un et al., 2009 |
| **Turkey** | 2002 | EU180616 | TR33 | human | Un et al., 2009 |
| **Turkey** | 2000 | EU180617 | TR40 | human | Un et al., 2009 |
| **Turkey** | 2001 | KP723631 | RV1138 | dog | This study(APHA) |
| **Turkey** | 2001 | KP723632 | RV1144 | fox | This study(APHA) |
| **Turkey** | 2000 | KP723633 | RV1145 | fox | This study(APHA) |
| **Turkey** | 2003 | KP723634 | RV1382 | dog | This study(APHA) |
| **UAE (Emirates)** | 1991 | KP723635 | RV XII | camel | This study (Vienna) |
| **UAE (Emirates)** | 1992 | KP723636 | RVXIII | camel | This study (Vienna) |
| **UAE (Emirates)** | 1994 | KP723637 | RVXIV | dog | This study (Vienna) |
| **Yakutia** | 1988 | AY352488 | na | Arctic fox | Kuzmin et al., 2004 |

na= data not available
