# Supplementary figures and images for: Complex Epidemiology of a Zoonotic Disease in a Culturally Diverse Region: Phylogeography of Rabies Virus in the Middle East
Source: PLoS Negl Trop Dis. 2015 Mar 26;9(3):e0003569. doi: 10.1371/journal.pntd.0003569 (PMC4374968; doi:10.1371/journal.pntd.0003569)

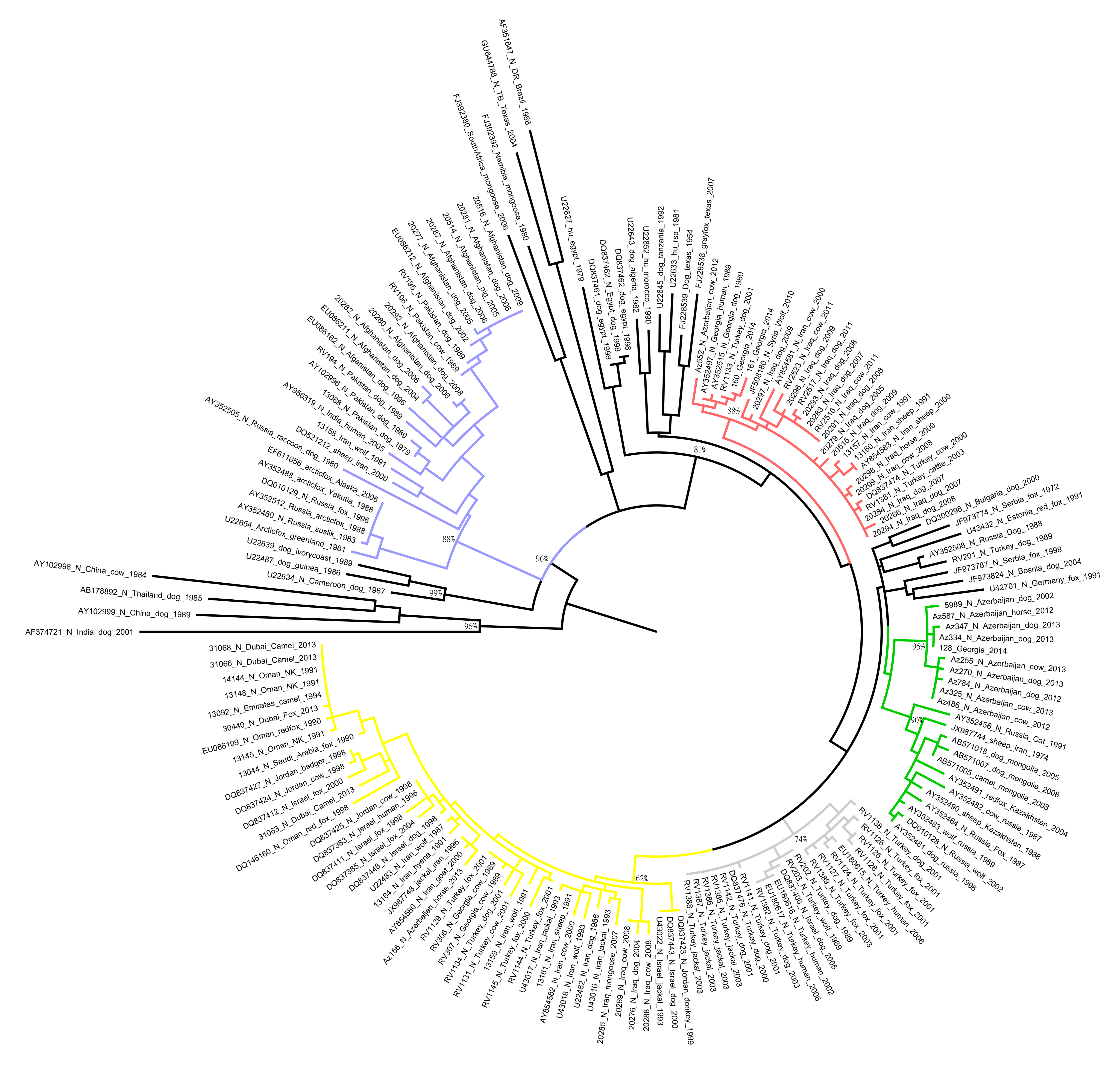

Supplement: S1 Fig — Bootstrap values are shown at selected key nodes. (TIF) [file pntd.0003569.s002.tif]

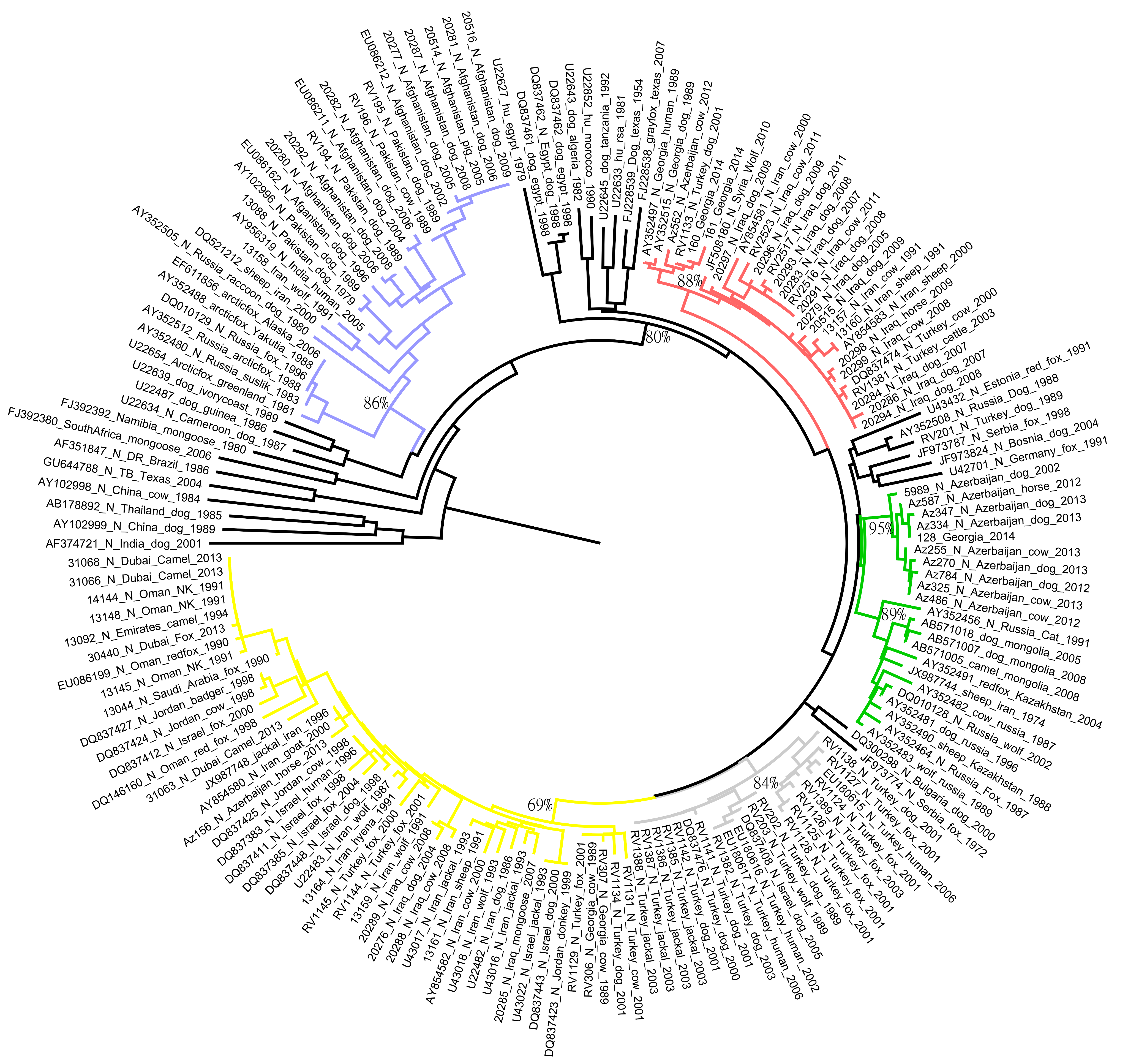

Supplement: S2 Fig — Boostrap values are shown at selected key nodes. (TIF) [file pntd.0003569.s003.tif]
